# New Drugs and Novel Cellular Targets against Tuberculosis

**DOI:** 10.3390/ijms232213680

**Published:** 2022-11-08

**Authors:** Giulia Degiacomi, Vadim Makarov, Maria Rosalia Pasca, Laurent Roberto Chiarelli

**Affiliations:** 1 Department of Biology and Biotechnology “Lazzaro Spallanzani”, University of Pavia, 27100 Pavia, Italy; 2 Research Center of Biotechnology RAS, 119071 Moscow, Russia

*Mycobacterium tuberculosis* (*Mtb*) is the etiological agent of tuberculosis (TB), one of the most life-threatening communicable diseases, which causes 10 million new cases each year and results in an estimated 1.4 million deaths globally [1]. The spread of drug-resistant *Mtb* strains is worrisome for not only the current antitubercular therapy, but also for drugs recently released on the market. Furthermore, the target of ending TB by 2030, as stated in the Sustainable Development Goals of the United Nations, will conceivably not be achieved, also due to the COVID-19 pandemic that has drained many resources from health care systems.

*Mtb* has evolved several strategies to escape bactericidal mechanisms put in place by the host immune system making it an extremely successful pathogenic bacterium. To fight the multifaceted strategies of *Mtb*, it is necessary to respond by applying effective and powerful solutions ranging from whole-cell screening to drug repurposing and to host-directed therapy. Research has a major role to play in finding new in vitro models to study and implement the development of new TB treatments, accelerating the discovery of novel drugs and new cellular targets towards *Mtb*.

This Special Issue focuses on the recent findings in TB drug discovery and provides an overview of the ongoing efforts to fight tuberculosis.

In the first research article of this Special Issue, Lara-Espinosa and co-worker investigated the antibacterial activity in lung and the anti-inflammatory effects in brain of curcumin, in a murine model of pulmonary TB [2]. Indeed, neuroinflammation and neuropsychiatric abnormalities have been already demonstrated in an experimental model of progressive pulmonary TB, which could explain the frequently noticed relationship between depression, anxiety, and TB [3]. The evidence that the treatment of infected animals with curcumin led to a reduction in the bacillary load in the lung, as well as a decreased neuroinflammation, suggest its possible use as an adjuvant in TB therapy [2].

Drug repurposing is a reasonable and wise alternative to the traditionally time and resource consuming de novo drug discovery programs, particularly for underfunded or neglected diseases. In this context, the work of Ezquerra-Aznárez and colleagues [4] demonstrated the antitubercular activity of avermectins, macrocyclic lactones already used in veterinary as anthelmintics. Another interesting feature of these compounds is that they likely have multiple targets in *Mtb*, including the well-known and promiscuous target DprE1 [4].

TB treatment, particularly for patients with drug-resistant form, is known to be a lengthy and complex regimen that requires the administration of numerous and potentially toxic antimicrobials. Such treatment conceivably can affect several processes, particularly of the immune system cells, thus mediating the host immune response to TB. Within the research article of Cahill et al. [5], the authors investigated the immunometabolic profile of stimulated human macrophages, upon exposure to the first-line and some second-line TB drugs. Indeed, they demonstrated that some antimicrobials are able to modulate host immune response, altering glycolysis, oxidative phosphorylation, and the secretion of cytokines and chemokines. These findings suggest that these effects can elicit host-directed functions, thus being beneficial even in patients with drug-resistant TB, highlighting the possibility of improving the use of long-established antimicrobials [5].

Certainly, the discovery of new antitubercular drugs with novel mechanisms of action still remains among the best weapon in the fight against multi-drug-resistant TB. The papers of Popovici et al. [6] and of Noschka and co-workers [7] presents two novel potential antitubercular compounds. The former article reports a classical drug discovery campaign, that led to the synthesis of new series of thiosemicarbazide derivatives displaying bacteriostatic activity against *Mtb*. The latter paper describes the characterization and the biological activity of a fragment Granulysin, an antimicrobial peptide active against fungi, parasite, and bacteria. Indeed, despite its potent activity, Granulysin presents some drawbacks such as large size, off-target effects, and high costs for manufacturing. In contrast, the fragment displays antimicrobial activity against *Mtb* and a panel of non-tuberculous mycobacteria, without significant off-target effects in both human cells and zebrafish model.

The articles that conclude this Special Issue are two reviews about the advancement in TB drug discovery. The first review of Egorova and colleagues [8] focalizes on the latent tuberculosis infection (LTBI), which represents another challenge in TB management. The review describes the characteristics of LTBI, the in vitro and in vivo models that have been developed to study dormant *Mtb*, as well as the alternatives for the treatment and the active compounds identified so far. The second review, by Ejalonibu and co-workers [9], describes the contribution of computational techniques in the development of novel anti-tuberculosis drugs, outlining the different approaches used and the main active compounds afforded, as well as the future opportunities given by the advancement in machine learning and artificial intelligence.

In summary, the Special Issue includes several articles that will hopefully be of interest to a wide audience, and which contribute to the fight against the worrisome plague of tuberculosis, raising awareness that resources and attention are still needed from global health systems and other stakeholders.
